# Advanced three-dimensional anatomical mapping of saphenous and inferior medial genicular nerve branching: enhancing precision in knee joint denervation

**DOI:** 10.1093/pm/pnae102

**Published:** 2024-10-17

**Authors:** Paula J Yu, Eldon Loh, Anne M R Agur, John Tran

**Affiliations:** Division of Anatomy, Department of Surgery, University of Toronto, Toronto, Ontario M5S 1A8, Canada; Department of Physical Medicine and Rehabilitation, Western University, London, Ontario N6C 0A7, Canada; Parkwood Institute Research, Lawson Health Research Institute, London, Ontario N6C 0A7, Canada; Division of Anatomy, Department of Surgery, University of Toronto, Toronto, Ontario M5S 1A8, Canada; Department of Physical Medicine and Rehabilitation, Western University, London, Ontario N6C 0A7, Canada; Parkwood Institute Research, Lawson Health Research Institute, London, Ontario N6C 0A7, Canada

**Keywords:** infrapatellar branch of saphenous nerve, inferior medial genicular nerve, radiofrequency ablation, anatomy

## Abstract

**Background:**

Radiofrequency ablation is a common non-opioid treatment to manage chronic knee pain. The inferior medial genicular nerve is conventionally targeted. It has been suggested that the infrapatellar branch (saphenous nerve) should also be targeted. There is controversy regarding the contribution of the infrapatellar branch to the innervation of the knee joint capsule.

**Objective:**

(1) Identify the frequency of the branching pattern(s) of the infrapatellar branch in three-dimensional (3D); (2) Assess spatial relationships of branches of infrapatellar branch to the inferior medial genicular nerve; (3) Determine if capturing infrapatellar branch could result in additional benefit to the existing protocol.

**Design:**

Anatomical study.

**Methods:**

The infrapatellar branch and inferior medial genicular nerve were serially dissected, digitized, and modelled in 3D in 7 specimens (mean age 91.3 ± 6.5; 2F/5M) and their relationship documented. The spatial relationship of the nerves was used to assess the anatomical efficacy of including the infrapatellar branch in the protocol.

**Results:**

The infrapatellar branch is most frequently a cutaneous nerve. This nerve was variable and found to be unbranched or have 2-3 branches and in all specimens was located superficial to the branches of inferior medial genicular nerve. When the infrapatellar branch (1) coursed more distally, the strip lesion would not capture the infrapatellar branch but would capture inferior medial genicular nerve consistently; (2) overlapped with the inferior medial genicular nerve, the strip lesion would capture both nerves.

**Conclusions:**

Proposed protocol targeting the infrapatellar branch is likely to capture the inferior medial genicular consistently regardless of the anatomical variation of the infrapatellar branch.

## Introduction

The knee joint is the most common joint affected by osteoarthritis (OA) representing 60.6% of cases globally between 1990 and 2019.^1^ Chronic pain of the knee joint, as with other articulations affected by OA, results in decreased participation “in meaningful activities, decreased well-being, and psychological distress.”^2^ Radiofrequency ablation (RFA) is a common non-opioid treatment option to manage OA-related chronic knee joint pain.^3^

Conventionally, nerves coursing on the medial aspect of the knee joint targeted by RFA techniques include the inferomedial genicular nerves (IMGN) and superomedial genicular nerves (SMGN),^4^ with several clinical studies reporting positive outcomes.^5–7^ However, Hu et al,^8^ Fonkoue et al,^9^ and Beckwith et al^10^ suggested that the infrapatellar branch of the saphenous nerve (IPBSN) should also be targeted in RFA protocols to treat medial knee joint pain. Each of the three studies proposed a “treatment line” to localize an RFA strip lesion to capture the IPBSN based on anatomical landmarks. Presently, there is a controversy regarding the contribution of the IPBSN to the innervation of the knee joint capsule. The IPBSN is classically described as a cutaneous nerve that supplies sensation to the skin of the inferomedial aspect of the anterior knee joint, however, it has been reported to have an articular branch in a small number of specimens.^11^ Additionally, in earlier literature, an articular branch was described to arise from the saphenous nerve.^12–14^

Previous anatomical literature have also described the detailed course of the IMGN.^11^^,^^14^ However, the three-dimensional (3D) anatomical relationship between the IPBSN and IMGN has not been investigated. Knowledge regarding the relationship between the IPBSN and IMGN is necessary to determine which nerves are potentially captured with the proposed RFA protocols. Therefore, the purpose of this cadaveric study was to (1) map in 3D the course of the IPBSN and IMGN and their terminal branches using 3D Cartesian coordinate data; (2) record and compare the sites of termination of the branches of IPBSN and IMGN using the 3D models and dissection photographs; and (3) based on results, evaluate the potential capture rate of the IPBSN and IMGN.

## Methods

Seven formalin embalmed cadaveric specimens (2F/5M) with a mean age of 91.3 ± 6.5 years old were used in this study. Inclusion criteria included no visible signs of injury, pathology, or previous surgery. The study was approved by the University of Toronto Health Sciences Research Ethics Board (Protocol #: 27210).

Following removal of the skin, the IPBSN and IMGN were traced and then digitized along with any visible bony landmarks using a Microscribe G2X Digitizer (Immersion Corporation, San Jose, CA, USA). The Microscribe digitization method enables the capture of 3D positional Cartesian coordinate data.^15^^,^^16^ The accuracy of the digitizer is ± 0.23 mm (manufacturer data), intra-rater reliability of 0.926 and inter-rater reliability of 0.989.^17^ In order to digitize the IPBSN and IMGN and their branches, short segments of each nerve were sequentially exposed by removing overlying tissue and then digitized at 1 mm intervals to their termination. The IPBSN was dissected first as it lies superficial to the IMGN. The IPBSN was first localized in the subcutaneous tissue as it pierced the crural fascia. Each branch of the IPBSN was traced to its termination by carefully removing surrounding subcutaneous tissue. The IPBSN was digitized throughout its course, simultaneously with bony landmarks. The site of termination of each branch was recorded.

To localize the IMGN, the sciatic nerve and the proximal part of the tibial and common fibular nerves were exposed from mid-thigh to proximal leg by removing all overlying tissue. The IMGN was localized as it branched from the tibial nerve and was joined by the inferior medial genicular artery and veins. Next, the medial head of gastrocnemius was reflected to reveal the underlying inferior medial genicular neurovascular bundle. The inferior medial genicular artery and veins were removed in short segments to expose the IMGN and its branches as they coursed anteromedially to lie deep to the medial collateral ligament (MCL). The middle third of the MCL was resected to enable the exposure of the part of the IMGN that lies deep to the MCL. All branches of the IMGN were traced to their termination in the anterior and medial aspect of the knee region. The IMGN and its branches were digitized concurrently with bony landmarks. The site of termination of each branch was recorded.

Each specimen was photographed throughout the dissection and digitization process. When the dissection was completed, the specimen was skeletonized, leaving the knee joint capsule intact. The skeletonized specimen was laser scanned using a Faro Laser ScanArm (FARO Technologies, Lake Mary, Florida, USA; accuracy ±35 mm).

The Cartesian coordinate data of the digitized nerves and anatomical landmarks, along with the laser-scanned data of the skeletal elements were imported and modelled in 3D using AutoDesk Maya (AutoDesk Inc., San Rafael, CA) with custom plug-ins developed in the laboratory. The 3D models included the course and distribution of the IPBSN and IMGN, skeletal and soft tissue landmarks, and the underlying skeleton including the femur, patella, tibia, and fibula. The models were used to visualize and document the course and distribution of the IPBSN and IMGN as in situ. Next, bony and soft tissue landmarks visible with ultrasound were identified that could be used to localize the IPBSN and IMGN.

The data were used to define the course of the branches of IPBSN and IMGN. The sites of termination of the branches of each nerve were compared using the 3D models and dissection photographs. Next, the role of IPBSN in RFA procedures was evaluated.

## Results

**Figure 1.**
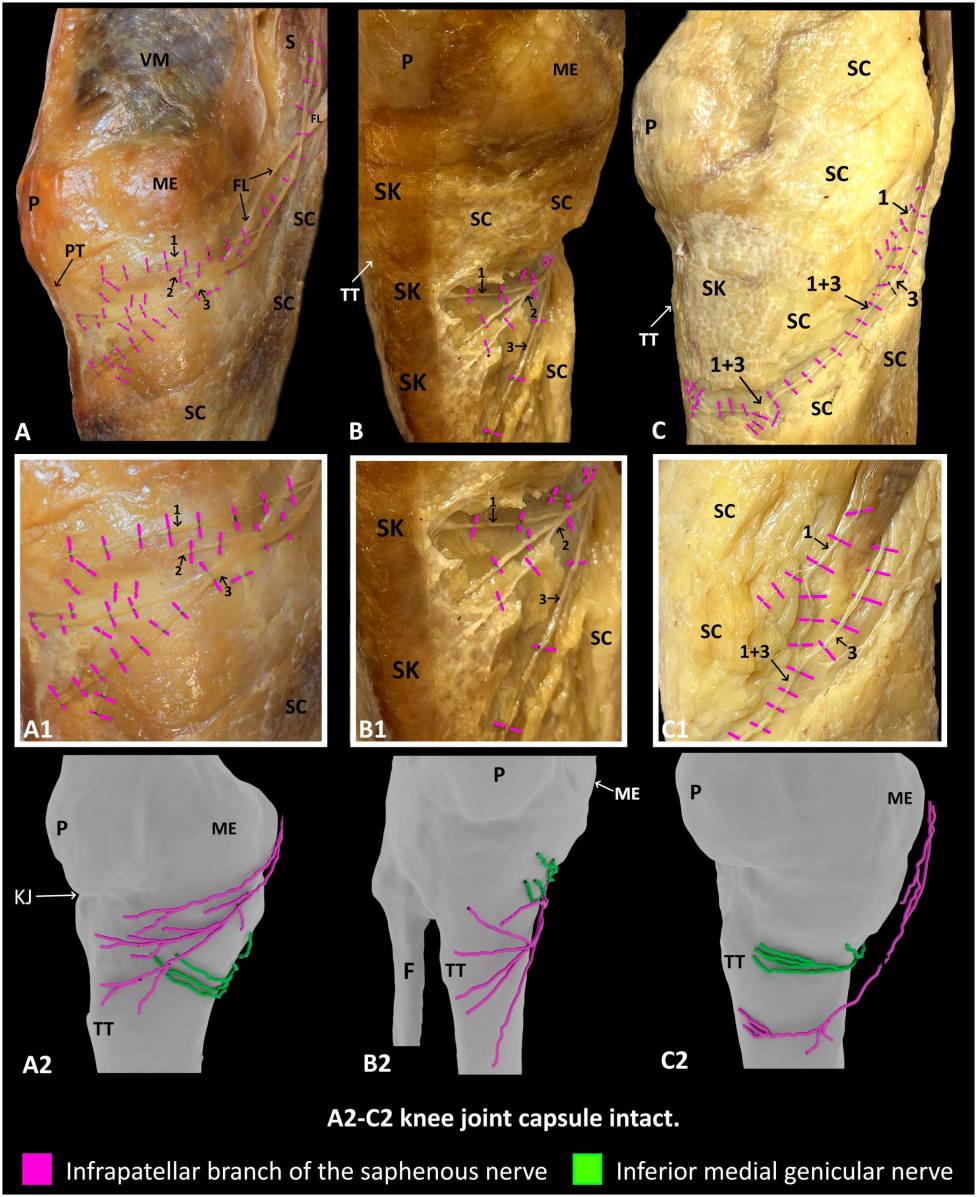
Dissection and 3D reconstruction of IPBSN and IMGN. (A-C) Dissections showing variable branching patterns of IPBSN relative to IMGN and bony landmarks. (A1-C1) Enlargement of the branches of IPBSN coursing in the subcutaneous tissue with no articular branches to the knee joint capsule. (A2-C2) 3D models showing the varying spatial relationship between IPBSN and IMGN. Branches of IPBSN: Superior (1), middle (2), inferior (3). F indicates fibula; FL, fascia lata; ME, medial epicondyle; KJ, knee joint; P, patella; PT, patellar tendon; S, sartorius; SC, subcutaneous tissue; SK, skin; TT, tibial tuberosity; VM, vastus medialis.

In this study, the IPBSN and IMGN were found in all specimens. The IPBSN originated from the saphenous nerve in the adductor canal in all specimens and then either pierced sartorius (*n* = 3; Figure 1A) or curved around its posterior border (*n* = 4; Figure 1C1) to penetrate the fascia lata. The IPBSN coursed anteromedially, and then divided into 2-3 branches (*n* = 6; Figure 1A and B) or remained unbranched in 1 specimen (Figure 1C). There was a superior, middle, and inferior branch in specimens with 3 branches (*n* = 3; Figure 1A1 and B1) and a superior and inferior branch in specimens (*n* = 3) with 2 branches. The branches were located proximally (*n* = 3; Figure 1A) with the superior branch coursing along the apex of the patella or distally with the superior branch coursing at the level of tibial tuberosity (*n* = 3; Figure 1B). If there were 3 branches, the superior branch coursed along the apex of patella, the middle branch coursed inferomedially to the medial border of the patellar tendon, and the inferior branch coursed further distally to terminate at the level of the tibial tuberosity (Figure 1A). In one specimen (with 3 branches), all of the branches coursed inferior to the tibial tuberosity (Figure 1B). If there were 2 branches, the superior and inferior branches arborized to cover the territory of the middle branch. In the case of no branching, a single IPBSN continued inferomedially to terminate inferior to the tibial tuberosity (Figure 1C). No articular branches originating from the IPBSN were found.

**Figure 2.**
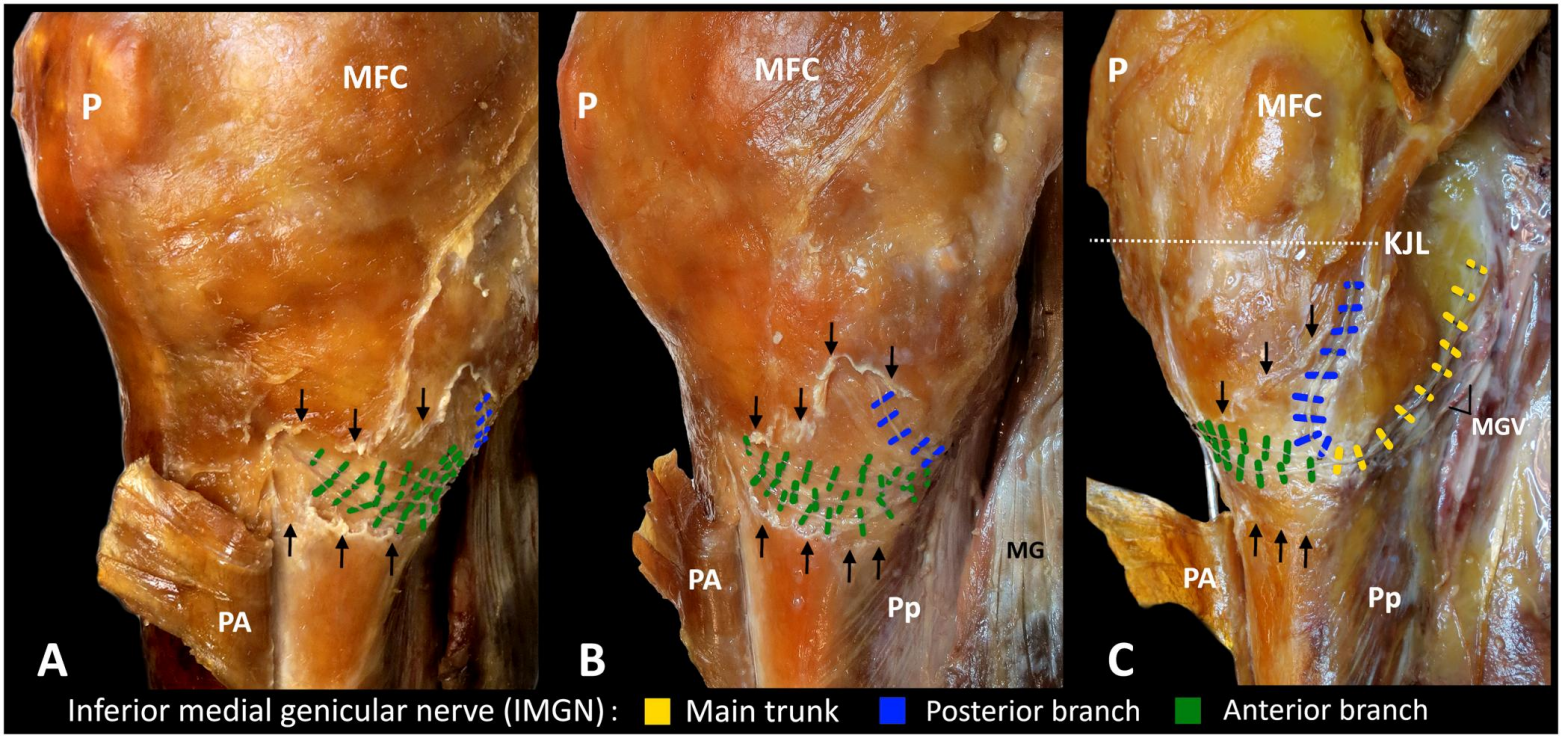
Dissection of the articular branches of IMGN. (A) Anteromedial view. (B) Medial view. (C) Posteromedial view. Black arrows indicate transection sites of the MCL of the knee; MFC, medial femoral condyle; P, patella; PA, pes anserinus; Pp, popliteus; MGV, medial genicular vessels; KJL, knee joint line; MG, medial head of the gastrocnemius.

**Figure 3.**
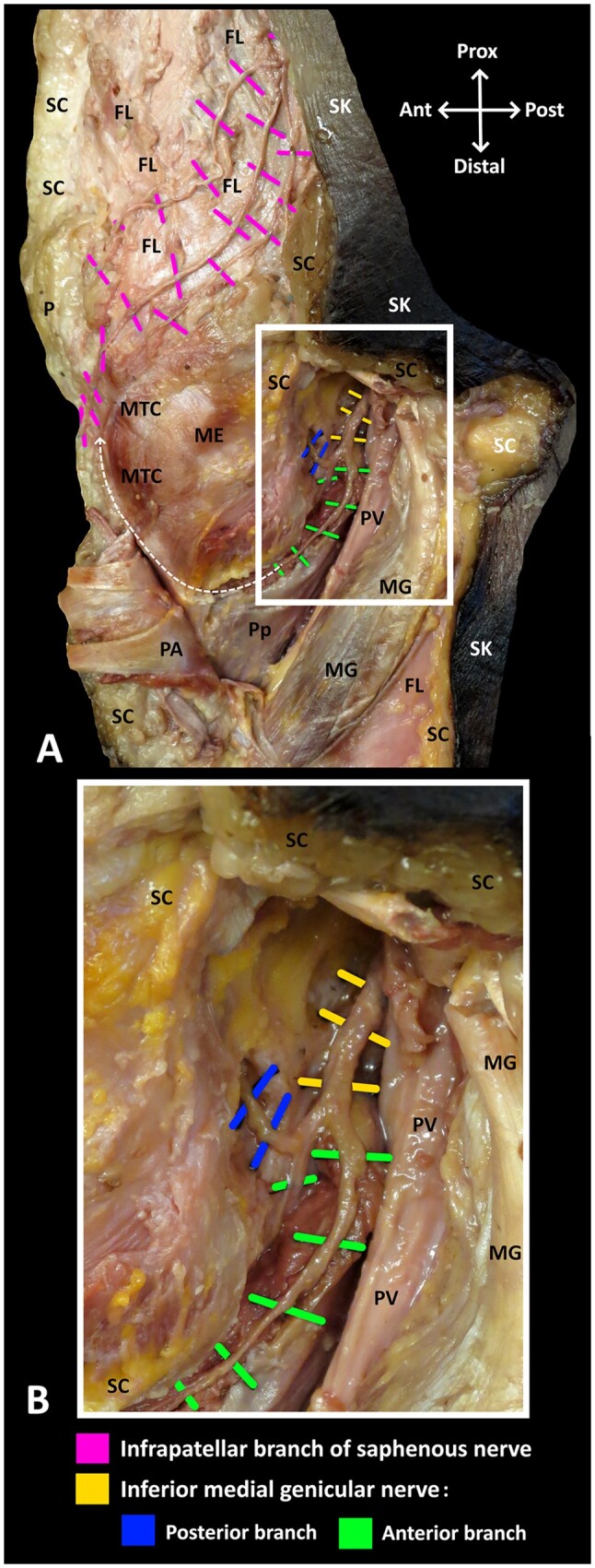
Course of the branches of IPBSN and IMGN. (A) Medial view. (B) Enlargement of articular branches of IMGN coursing deep in the popliteal fossa. White dashed line arrow indicates the course of the anterior branches of IMGN. PV, popliteal vessel; FL, fascia lata; SC, subcutaneous tissue; SK, skin; Pp, popliteus; PA, pes anserinus; MG, medial head of gastrocnemius; MTC, medial tibial condyle; ME, medial epicondyle.

In all specimens, the IMGN branched from the tibial nerve in the popliteal fossa deep to the medial head of gastrocnemius (Figure 2B and C). The IMGN coursed obliquely, inferomedially along the superior border of popliteus with the inferior medial genicular vessels to lie on the medial surface of the tibia (Figure 2C). Next, the IMGN coursed deep to the MCL, where it branched into multiple anterior branches and a single posterior branch (Figure 2B). In 6 specimens, both the anterior and posterior branches coursed deep to the MCL to terminate in the knee joint capsule (Figure 2). In 1 specimen, the posterior branch of the IMGN terminated in the knee joint capsule posteromedially and did not course deep to the MCL (Figure 3). The anterior branches were located inferior to the posterior branch in all specimens (Figures 2 and 3). The anterior branches of the IMGN coursed transversely around the proximal tibial metaphysis inferior to the medial tibial condyle and then continued anterosuperiorly to terminate in the inferomedial aspect of the anterior knee joint capsule (Figure 2A and B). The posterior branch coursed superomedially from its bifurcation on the periosteum of the tibia to terminate in the posteromedial capsule (Figure 2C).

The IPBSN coursed superficially, whereas the IMGN lay at a considerably deeper plane when it emerged from the popliteal fossa (Figure 3). The IPBSN coursed anteromedially in the subcutaneous tissue to terminate in the skin with no articular branches (Figures 1A-C and 3A). The anterior branches of the IMGN, which are conventionally targeted in RFA procedures, terminated in the inferomedial aspect of the anterior knee joint capsule (Figures 2A and B and 3A). In this location, the branches of IPBSN lay either immediately superficial to the anterior branches of the IMGN in 4 specimens (Figures 1A2 and 3A) or inferior to the articular branches of the IMGN in 3 specimens (Figure 1B2 and C2).

## Discussion

In this cadaveric study, 3D modelling technology was used to record and compare the course and termination sites of IPBSN and IMGN innervating the medial knee joint. Clinically, understanding which sensory nerves innervate the knee joint capsule and their spatial relationships provides an anatomical foundation for the development of evidence-based RFA protocols to treat OA-related chronic knee pain.

The IPBSN, in this study, was found to have variable branching patterns, consistent with previous publications.^18–20^ The IPBSN was described to divide into 2 branches (superior, inferior),^18^ 3 branches (superior, middle, inferior),^19^ or remain unbranched.^20^ Kalthur et al^20^ also reported the frequency of the branching patterns of IPBSN and described the unbranched type of IPBSN as the most common. However, in this study, the IPBSN most often divided into 2 or 3 branches. Furthermore, both Tennent et al^19^ and Kalthur et al^20^ reported that the branches of IPBSN were mostly distributed between the patella and tibial tuberosity. The findings of this study corroborated with previous literature,^18–20^ where the branches of the IPBSN were found to course between the apex of the patella and the tibial tuberosity. In 3 specimens, the branches of IPBSN coursed inferior to the tibial tuberosity. In contrast, the course of the IMGN was found to be consistent with previous literature,^11^ where in all specimens, the nerve coursed along the superior border of the popliteus before giving off the anterior and posterior branches.

In the previous literature, there were inconsistent findings regarding the termination sites of the IPBSN.^11^^,^^14^^,^^21–23^ Gardner^14^ and Fonkoué et al^23^ reported the presence of articular branches (frequency not stated) originating from the IPBSN. In an earlier study done in our laboratory, Tran et al^11^ found articular branches from the IPBSN in 3 out of 15 specimens. Most studies including this study have not found an articular branch and described the IPBSN as a cutaneous nerve innervating the anterior knee joint.^20–22^ Additionally in the literature, it has been suggested that targeting the IPBSN could be useful to treat chronic post-surgical neuralgia with a cutaneous distribution.^24^ As the IPBSN lies in the subcutaneous tissue immediately deep to the skin, the RFA lesion could result in internal skin burn.^25^

The course and termination sites of IMGN, in this study, concur with previous literature. Both Gardner^14^ and Tran et al^11^ described the IMGN as an articular nerve coursing deep to the MCL along the periosteum to terminate in the inferomedial aspect of the anterior knee joint capsule. The IMGN was present in all specimens in the current study and in the previous studies.

**Figure 4.**
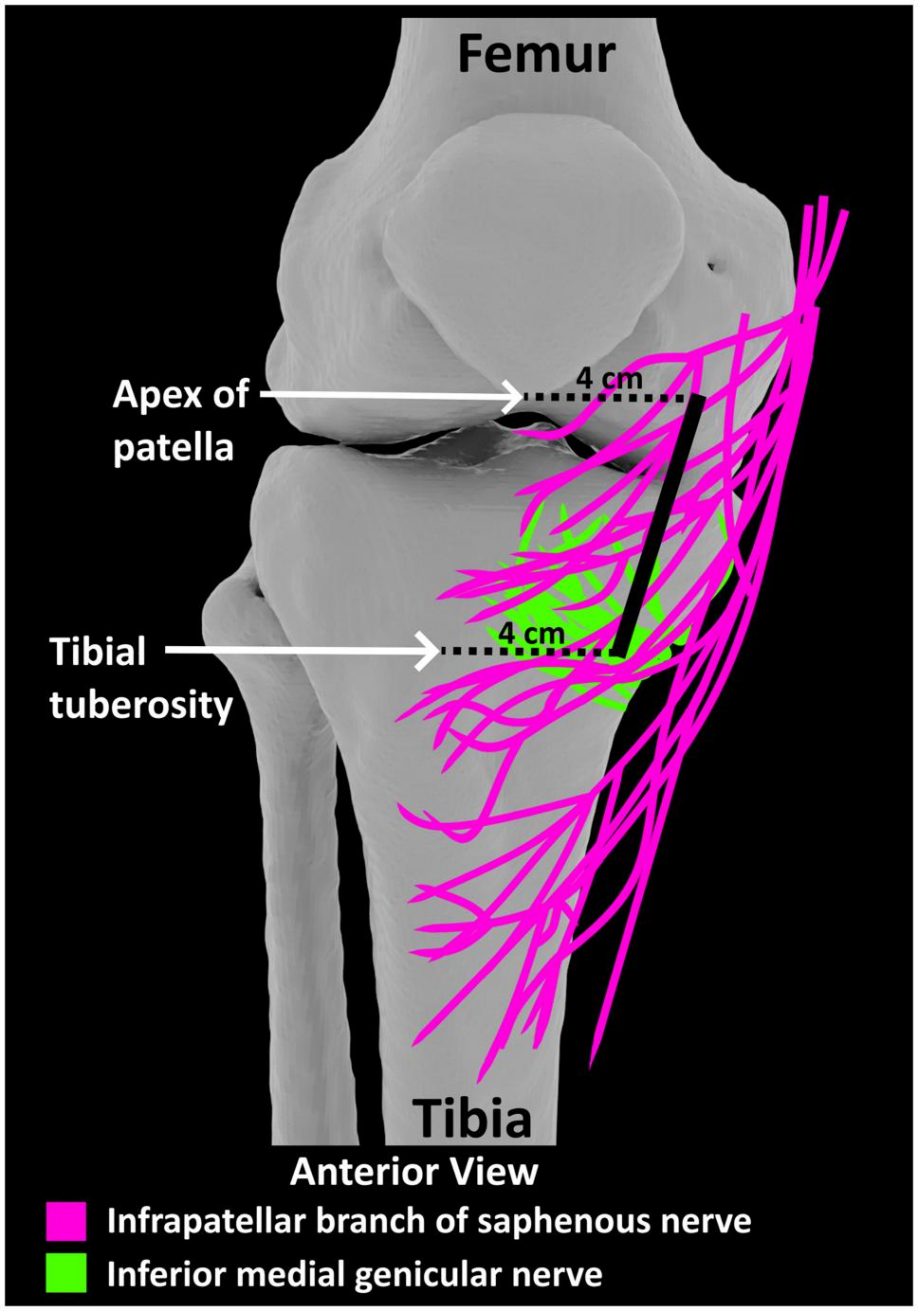
Frequency map of the branches of IPBSN and IMGN in relation to the proposed IPBSN treatment line (black line). Treatment line is 4 cm from the apex of patella and tibial tuberosity (Fonkoue et al, Beckwith et al).

The results of the current anatomical study have possible significant clinical implications. Several recent publications have recommended IPBSN as a suitable nerve target of RFA procedures to treat OA-related chronic knee pain.^8–10^^,^^26^^,^^27^ Based on our findings in the current study and previous studies,^11^^,^^20–22^ it was observed that the IPBSN is most often a cutaneous nerve suggesting that lesioning the IPBSN may not relieve chronic knee pain caused by OA. Based on a case report,^28^ a skin burn was reported in a patient with little subcutaneous tissue when an RFA lesion of the IMGN was performed. It was suggested that the amount of subcutaneous tissue could affect the outcome of the RFA lesion. The RFA strip lesion proposed by Hu et al, Fonkoue et al, and Beckwith et al targeting the IPBSN is likely to capture the IMGN consistently regardless of the anatomical variation of the IPBSN (Figure 4). The anatomical variation of the IPBSN has implications on its capture rate when using the proposed RFA protocol. When the IPBSN coursed more distally, the proposed RFA strip lesion would not capture the IPBSN but would capture the IMGN consistently (Figure 4). When the IPBSN overlapped with the IMGN, the proposed RFA strip lesion would capture both nerves. This finding suggests any potential clinical benefit from this proposed protocol may be due to the consistent capture of the underlying IMGN supplying the knee joint capsule. In a future study, the results of this study can be used to further assess the anatomical efficacy of the RFA strip lesion previously proposed by Hu et al,^8^ Fonkoue et al,^9^ and Beckwith et al^10^ to determine possible optimal lesion site(s).

A limitation of this study is the small sample size. This is due to the time-consuming nature (40-50 hours per specimen) of the dissection of the fine articular branches to their termination. Since this is an anatomical study, further clinical investigation is needed to determine the analgesic effectiveness of targeting IPBSN and IMGN.

## Conclusions

In conclusion, the IMGN coursed deep to the IPBSN and consistently innervated the medial knee joint capsule in all specimens. The IPBSN had variable branching patterns. The proposed RFA protocol, intended to target the IPBSN only, will also capture the IMGN in all specimens as it courses to the medial knee joint capsule. Moreover, the IPBSN did not innervate the knee joint capsule and may not be an ideal nerve target of RFA procedures to treat chronic knee pain caused by OA-related pathologies.
